# Construction and Development of an Enhanced Recovery After Surgery Program for the Surgical Management of Patients With Spinal Metastasis: A Modified Delphi Study

**DOI:** 10.1111/os.14375

**Published:** 2025-01-23

**Authors:** Fanjie Li, Wenlong Yu, Haohan Zhou, Fan Zhang, Zihuan Zhou, Qiang Gao, Xin Gao, Luosheng Zhang, Yinjie Yan, Quan Huang, Xinghai Yang, Peilin Chu, Mengchen Yin

**Affiliations:** ^1^ Department of Orthopaedics Maanshan General Hospital of Ranger‐Duree Healthcare Anhui China; ^2^ Department of Orthopaedics, Longhua Hospital Shanghai University of Traditional Chinese Medicine Shanghai China; ^3^ Department of Orthopedics Oncology, Changzheng Hospital Second Affiliated Hospital of Naval Medical University Shanghai China

**Keywords:** Delphi study, development, enhanced recovery after surgery, spinal metastasis, surgical safety checklist

## Abstract

**Objective:**

As an effective treatment for spinal metastasis (SM), ERAS protocol can significantly reduce the length of hospital stay and complications in patients. Establishing an ERAS program for perioperative care after SM surgery is a clinical problem that needs to be addressed urgently. We aimed to develop an Enhanced Recovery After Surgery (ERAS) program and Surgical Safety Checklist (SSC) that conferred clinical benefit to patients with SM and made it relatively easy to manage the condition. We believe that our findings could help establish and promote the continuous improvement of additional ERAS programs for SM.

**Methods:**

This is a modified Delphi study. We used a two‐round process using data acquired from a review of relevant literature and involving a multidisciplinary panel of experts from different hospitals in China. The modified Delphi survey was conducted from February 1, 2024 to April 20, 2024. The experts were invited to evaluate each of the current relevant ERAS recommended topics to determine the appropriateness of inclusion in the ERAS program and SSC with the 5‐point Likert scale. Used the results to create specific ERAS and SSC (70% consensus) programs. Close to consensus (65%–69% consensus) findings were considered for the follow‐up survey.

**Results:**

A multicenter, multidisciplinary group of physicians (*n* = 75), including clinical workers, researchers, anesthesiologists, nursing specialists, psychologists, nutritionists, and caregivers, with experience in managing patients with SM, were asked to participate. Using the modified Delphi process, we arrived at a consensus for the ERAS program. This included 37 consolidated items in the domains in the following order: preoperative, intraoperative, postoperative, and discharge. The SSC included 37 consolidated items in the domains in the following order: before the induction of anesthesia, before skin incision, and before the patient leaves the operating room.

**Conclusion:**

This study, based on the modified Delphi process, helped us develop ERAS and SCC consensus‐driven best practice recommendations, including suggestions related to perioperative anesthesia, surgery, and nursing for SM. We hope that this study, in which we integrated both traditional Chinese and Western medical treatment protocols, can provide a basis for a rapid rehabilitation program for surgical interventions in SM.

## Graphical abstract

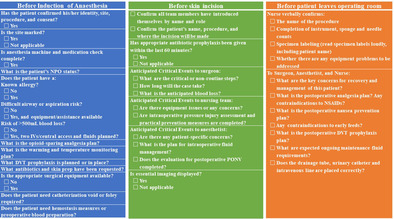

**Abbreviations**
ERAS: Enhanced Recovery After Surgery Program
SM: spinal metastasis
SSC: Surgical Safety Checklist
TCM: traditional Chinese medicine

## 1 Introduction

The treatment for spinal metastasis (SM) has remained a challenge [1, 2, 3]. Surgical treatment is undoubtedly effective for relieving symptoms and improving the quality of life of patients [4, 5, 6]. The intraoperative and postoperative complications of patients must be reduced, and their postoperative survival rate should be improved. Enhanced Recovery After Surgery (ERAS) is a multimodal perioperative care pathway designed to achieve early recovery for patients undergoing surgery [7]. The ERAS program reduces the length of stay and postoperative complications in patients [8]. Increased compliance with the ERAS program is also associated with prognosis. To our knowledge, ERAS programs for SM have not been reported in any study. Therefore, establishing an ERAS program for perioperative care after SM surgery is a clinical problem that needs to be addressed urgently.

The shortcomings of existing ERAS programs are well known. When ERAS programs were first established, the preoperative psychological or physiological status of patients was not assessed, and the importance of safety checks for the surgery and comprehensive treatment of patients after surgery was not considered [9, 10, 11, 12]. The Surgical Safety Checklist (SSC) is a proven tool for optimizing perioperative patient outcomes [13]. Studies have shown that the use of ERAS programs and SSC can improve the degree of compliance with ERAS evidence‐based principles and maximize the efficacy of SSC and ERAS recommendations [14, 15].

Traditional Chinese medicine (TCM) also conforms to the concept of ERAS in tumor treatment. This mode of treatment can not only help kill tumor cells directly but also help enhance the sensitivity of modern medical therapy, reduce the toxic and side effects of such therapeutic procedures, and prevent tumor recurrence and metastasis. It plays a specific role in improving the preoperative nutritional status, decreasing pain, and treating tumor‐related depression and fatigue in patients. It can reduce the occurrence of postoperative complications [16, 17, 18, 19, 20, 21, 22]. The integration of TCM and the ERAS program is an important method to utilize the advantages of TCM.

Here, we report the exploratory phase of an ERAS project for identifying the key interventions of the ERAS programs for SM. We propose to: (i) incorporate TCM treatments with Chinese medical characteristics into ERAS programs; (ii) develop the ERAS program and SSC that conferred clinical benefit to patients and made the condition relatively easy to manage; and (iii) help establish and promote the continuous improvement of additional ERAS programs for SM.

## 2 Methods

To develop an ERAS and SSC scheme outlining the ERAS programs for SM, we used a two‐round modified Delphi process informed by a review of relevant literature and involving a multidisciplinary panel of experts from different hospitals in China.

### 2.1 Design

The Delphi process comprises a series of questionnaires that participants answer based on their expertise. It is an iterative, anonymous, group‐based process for gathering relevant opinions on a topic and eventually arriving at a consensus. When responding to the questionnaire, each participant can express their views and compare them with those of others. The participants are then allowed to respond a new set of questionnaires based on the revised views. The process continues until participants arrive at a consensus or a pre‐determined number of rounds are completed. A modified Delphi process also includes face‐to‐face meetings of participants [23].

The modified Delphi method was deemed appropriate for this study. When a strategy to incorporate TCM into ERAS programs for SM is being developed, several multidisciplinary participants can provide a wide range of opinions from their perspectives. We advised participants to make optimizations and suggestions based on these items. We did not provide recommendations on the SSC for SM surgery because we did not retrieve relevant literature. We used the SSC launched globally by the World Health Organization (WHO) in 2009 and combined it with the ERAS scheme. The final ERAS program and SSC were obtained using the modified Delphi method, which included one round of virtual meetings and two rounds of questionnaires [13].

### 2.2 Phase 1: Participatory Workshops

We invited authoritative clinical workers, researchers, anesthesiologists, nursing specialist, psychologists, nutritionists, and caregivers, with experience in managing patients with SM. We also accepted referrals to identify respondents for this stage. In the recruitment process, we took measures to minimize the inclusion of individuals from the same medical facility or geographic region. This approach effectively reduces potential bias related to participants' origins, such as different levels of medical institutions. After we identified quality ERAS items from the literature, we conducted participatory virtual workshops with the participants. The respondents were asked to reflect on the quality items for inclusion in a subsequent survey with a larger pool of participants. The participants were tasked with evaluating the suitability, feasibility, and compliance with the original ERAS program and the current WHO SSC for SM surgery.

We recorded detailed notes during the meeting. The recorded content included the evaluation and suggestions for each item. Based on feedback from the meeting, we prioritized each project and made appropriate modifications and integrations into the second phase of the survey.

### 2.3 Phase 2: Delphi Surveys

In this phase of the study, we included clinicians, anesthesiologists, and nursing experts from hospitals at all levels in China. The criteria for participating in the survey included at least 10 years of experience since qualification. We recruited experts through snowball sampling. We established no prescribed quota. We shared invitations to the potential participants sent via email. We provided a maximum of two reminders sent after the initial contact per round. We did not offer any incentives or compensation to the respondents. All respondents who completed the first round and provided valid contact information was first round was completed. Survey data from participants who completed the first round of the process but did not provide valid contact information were included in the analysis, but these experts were not invited to participate in subsequent rounds.

We conducted a two‐round modified Delphi process survey based on questionnaires and additional text explanations [24]. We followed the Standards for Quality Improvement Reporting Excellence 2.0 (SQUIRE 2.0) reporting guidelines [25, 26]. Consensus was defined by at least 70% agreement among experts. The experts were invited to evaluate each of the current relevant ERAS recommended topics (which were merged when duplication was found) to determine the appropriateness of inclusion in the ERAS program and SSC with the 5‐point Likert scale: “strongly agree,” “agree,” “neither agree nor disagree,” “disagree,” or “strongly disagree”. We used the results to create specific ERAS and SSC (70% consensus) programs. Close to consensus (65%–69% consensus) findings were considered for the follow‐up survey. There were no missing inclusion data, since respondents were encouraged to complete each topic before they progressed within the survey. Participating experts who completed the first round were also invited to evaluate the newly generated ERAS program and SSC. Experts could choose to recommend items for inclusion or exclusion in the ERAS program and SSC. In the second round, participant experts who completed the first round were invited to evaluate the novel ERAS and SSC prompts and the original prompts. The level of agreement from round 1 was indicated, and the experts could suggest including or excluding the topic‐derived prompt. Free‐text comments were again requested. Consensus to include was an unweighted summation of the following instances: include the item as proposed, include it but modify content, and include but move. The suggestions of the respondents to either modify the content or move the recommendation to another section of the ERAS and SSC were considered alongside the free‐text comments provided.

### 2.4 Consensus Meeting

An online interactive consensus meeting was conducted using the Tencent Conference platform. The consensus meeting was conducted in Chinese and chaired by an experienced independent facilitator. In the virtual convention, a selection of experts reviewed the qualitative results and summative quantitative review of the provided comments. A consensus was arrived at, and an ERAS program and SSC were produced. To make the consensus more objective, in the consensus meeting, the experts also revisited the quality items for which there was no consensus on high or low priority.

## 3 Results

### 3.1 Participants

A multicenter, multidisciplinary group of physicians (*n* = 75), including clinicians (*n* = 20), researchers (*n* = 15), anesthesiologists (*n* = 15), nursing specialist (*n* = 15), psychologists (*n* = 5), and caregivers (*n* = 5), with experience in managing patients with SM were asked to participate. Individuals were selected based on prior collaboration, clinical experience, and volume, prior research experience in SM, and membership in academic organizations. The occupation, workplace and city of the participants were used as Table S1.

### 3.2 Literature Review

The modified Delphi process included the identification of the existing ERAS programs through a preliminary literature review. The literature was systematically reviewed with adherence to the Preferred Items for Systematic Evaluation and Meta‐Analysis (PRISMA) statement guidelines [27, 28]. The principal literature search was conducted using MEDLINE, Embase, CNKI, and Cochrane databases to identify contributions related to the topic published. The keywords included “spinal metastasis,” “metastasis,” “enhanced recovery after surgery,” and “surgical safety checklist.” We checked the reference lists of all eligible articles for other relevant studies. We included systematic reviews, RCTs, and observational cohort studies that reported spinal surgery related to the ERAS topics. Our group provided participants with a list of existing ERAS programs, which included 19 ERAS items that had been implemented previously.

### 3.3 Delphi Survey Process of the ERAS Program

Based on the working group's recommended classification framework and literature review for ERAS program, we categorized the 39 items into the following four domains: preoperative (13 items), intraoperative (11 items), postoperative (10 items), and discharge (five items).

After review and confirmation by the steering committee, we prepared a list of 39 items and used it for the first round of the modified Delphi survey. The first round of the online modified Delphi survey was conducted from February 1 to February 15, 2024. All participants (*n* = 75, 100%) completed the questionnaires in this process. The work group received and reviewed 75 comments on the questionnaire entries. In the domain of “preoperative,” 25 comments (33.3%) suggested that “preoperative position training” and “primary tumor treatment” should be removed on the grounds. In the domain of “intraoperative,” 12 comments (16.0%) suggested that “surgical considerations” and “restrict placement of surgical site” should be removed. In the domain of “postoperative,” 19 comments (25.3%) suggested that “ambulation” should be removed. In the domain of “discharge,” 12 comments (16.0%) suggested that “patient evaluation” should be removed.

The second round of the Delphi survey was conducted from March 1 to March 15, 2024. All participants (*n* = 75, 100%) completed the questionnaires in this process. Eighteen comments (24.0%) suggested that “psychological evaluation,” 22 comments (29.3%) suggested that “temperature management,” and 32 comments (42.7%) suggested that “treatment of bloating and constipation” and “external use of Chinese medicine and fumigation therapy” should be added to the ERAS program.

The consensus meeting was held on April 20, 2024 in Shanghai, China. Under the auspices of the expert committee, the items were discussed in the following order of the domains: preoperative, intraoperative, postoperative, and discharge. The complete ERAS program items assessment process is shown in Table 1. The final ERAS program and the detailed definitions are provided in Table 2.

**TABLE 1:** Summary of the Delphi surveys process of ERAS protocol.

ERAS items	Delphi round 1	Delphi round 2	Consensus meeting
Preoperative			
Preoperative patient and family counseling and education	Include	Include	Include
Patient evaluation	Include	Include	Include
Psychological evaluation	—	Include	Include
Smoking and alcohol abstinence	Include	Include	Include
Nutritional intervention	Include	Include	Include
Antithrombotic prophylaxis	Include	Include	Include
Preoperative bowel intervention and bowel preparation	Include	Include	Include
Preop fasting and oral carbohydrates	Include	Include	Include
Pain management	Include	Include	Include
Hemostasis and prevent bleeding	Include	Include	Include
Preoperative anemia treatment	Include	Include	Include
Preoperative position training	Include	Exclude	—
Respiratory function training	Include	Include	Include
Primary tumor treatment	Include	Exclude	—
Intraoperative			
Surgical considerations	Include	Exclude	—
Anesthesia management	Include	Include	Include
Pressure injury prevention	Include	Include	Include
Temperature management	—	Include	Include
Fluid management	Include	Include	Include
Prophylactic antibiotics	Include	Include	Include
Wound suture	Include	Include	Include
Local incision anesthesia	Include	Include	Include
Restrict placement of surgical site drains	Include	Exclude	—
Preventing PONV	Include	Include	Include
Airway management	Include	Include	Include
Blood transfusion	Include	Include	Include
Postoperative			
Discontinuation of the urinary catheter and drainage	Include	Include	Include
Treatment of bloating and constipation	—	Include	Include
Postoperative delirium treatment	Include	Include	Include
Postoperative Chinese medicine treatment	Include	Include	Include
Postoperative acupuncture treatment	Include	Include	Include
External use of Chinese medicine and fumigation therapy	—	Include	Include
Ambulation	Include	Exclude	—
Pain management	Include	Include	Include
DVT prophylaxis	Include	Include	Include
Diet	Include	Include	Include
Postoperative PONV treatment	Include	Include	Include
Psychological evaluation	Include	Include	Include
Discharge			
Pain management	Include	Include	Include
Diet	Include	Include	Include
Ambulation	Include	Include	Include
Patient evaluation	Include	Exclude	—
Systemic audit	Include	Include	Include

**TABLE 2:** Detailed definitions of ERAS protocol items.

Items	ERAS program
Preoperative	
Preoperative patient and family counseling and education	Receive consultation from patients and their families; inform them of the relevant ERAS process; sign informed consent; introduce the concept of fast recovery after surgery, discharge standards.
Patient evaluation	Assess the patient's preoperative status using a series of scales, such as KPS, VAS, NRS 2002 score, VTE Caprini risk assessment scale, and Autar DVT risk assessment scale, PONV simple risk assessment scale.
Psychological evaluation	Assess the patient's psychological status (HADS score) before surgery and intervene selectively, and selective intervention is carried out through psychological counseling or drug treatment.
Smoking and alcohol abstinence	Abstinence smoking and alcohol for 2 weeks.
Nutritional intervention	Nutritional consultation to keep body mass index between 18.5 and 24, serum albumin level ≥ 3.5 g/dL.
Antithrombotic prophylaxis	Active/passive limb movement, graduated compression stockings, ICP pump, stopping antivascular endothelial growth factor‐targeted medications.
Preoperative bowel intervention and bowel preparation	Defecation induction with glycerine enema for chronic constipation or no defecation ≥ 2 days; bowel preparation is required for reoperation of sacral or pelvic tumors.
Preop fasting and oral carbohydrates	Patients without gastrointestinal motility disorders fast for 6 h before surgery and fast for 2 h before surgery; carbohydrate drinks are allowed within 2 h before surgery, unless the risk of aspiration is high within 4 h before surgery.
Pain management	Personalization of perioperative analgesia protocols, oral celecoxib, pregabalin, or tramadol.
Hemostasis and prevent bleeding	Different drugs need to be withdrawn at different time intervals before spinal surgery; intraoperative application of hemostatic drugs such as tranexamic acid.
Preoperative anemia treatment	Preoperative treatment of anemia by blood transfusion + recombinant human erythropoietin; maintenance of platelet counts at 50 × 10^9^/L and if not necessary, embolization was not chosen.
Respiratory function training	Assess the patient's lung function through pulmonary function tests, conduct pulmonary function exercises, and combine nebulization inhalation to improve the patient's lung condition.
Intraoperative	
Anesthesia management	Combined intravenous inhalation anesthesia, induced with propofol, sufentanil, and rocuronium, and maintained with propofol, fentanyl, and sevoflurane.
Pressure injury prevention	Preoperative stress injury risk assessment using a stress injury risk scale, and taking corresponding measures based on the assessment results, including reducing friction and shear forces, using pressure‐reducing tools, skincare, and regularly observing the location.
Temperature management	Constant body temperature monitoring throughout the surgery and actively attempt to keep the temperature above 36°C using forced‐air and electric heating pad þ warmed fluid irrigation and infusion.
Fluid Management	Use GDPFR strategy to guide perioperative fluid therapy and maintain isovolemia, non‐invasive cardiac output monitoring should be implemented, and appropriate adjustments should be made based on urine output, intraoperative blood loss, and hemodynamic parameters; maintain warm fluid infusion.
Prophylactic antibiotics	Prophylactic antibiotics should cover *Staphylococcus aureus* and should be given 30 min before surgery and again if surgery exceeds 3 h.
Wound suture	Absorbable suture for dura, muscle, and subcutaneous tissue, intradermal suture for skin incision.
Local incision anesthesia	Subcutaneous anesthesia before incision and wound suturing.
Preventing PONV	Choose combined general anesthesia to reduce the use of opioids; accurately control the operation time and shorten the anesthesia time.
Airway management	Glucocorticoids, such as methylprednisolone (20–40 mg) or hydrocortisone (100 mg), be administered intravenously before endotracheal intubation under general anesthesia to prevent intraoperative bronchospasm, throat complications, and potential allergic reactions; strictly follow the indications for endotracheal extubating after surgery, especially for patients undergoing upper cervical spine surgery.
Blood transfusion	When Hb is between 70 and 100 g/L, the decision on whether to transfuse should be made based on a comprehensive consideration of factors such as the patient's age, amount of bleeding, bleeding rate, cardiopulmonary function, and the presence or absence of hypoxia symptoms.
Postoperative	
Discontinuation of the urinary catheter and drainage	Early discontinuation of urinary catheters and drainage; when the drainage flow is < 50 mL at 24 h after closing the negative pressure; for patients with postoperative cerebrospinal fluid leakage whose fascial layer suture tightness is unknown, the retention time of the drainage tube should be extended and negative pressure drainage should be prohibited at the same time.
Treatment of bloating and constipation	Physical therapy combined with Chinese medicine or acupuncture can promote gastrointestinal motility and warm saline enema can be performed if necessary.
Postoperative delirium treatment	Pain relief, correction of anemia, restoration of circadian rhythm, correction of electrolyte imbalance, improvement of oxygenation level, increase of cerebral blood perfusion, and discontinuation of drugs that may cause delirium; dexmedetomidine can also be used to treat postoperative delirium.
Postoperative Chinese medicine treatment	Oral Chinese medicine after surgery improves nutritional status and appetite; enhancing damaged immunity and participates in tumor immunotherapy.
Postoperative acupuncture treatment	Reduce cancer pain, relieve cancer fatigue; treat postoperative insomnia, anxiety, and PONV.
External use of Chinese medicine and fumigation therapy	Local analgesia, reducing bleeding; preventing postoperative abdominal distension; reducing ascites in patients with liver cancer, stomach cancer, etc.
Pain management	Pain VAS score < 4, no analgesia or minimal oral non‐opioid medication; pain VAS score 4–6, oral or intravenous non‐opioid medication; pain VAS score > 7, opioid PCA.
DVT prophylaxis	Patients should undergo physical prevention of DVT after surgery and use anticoagulants, if necessary, based on coagulation function monitoring; for individuals at moderate and high risk of arterial thromboembolism, a combination of medication and physical prophylaxis is advised.
Diet	Light diet for 6 h after surgery, 8 h after surgery according to patient tolerance; semi‐liquid/solid diet for 12–24 h after surgery; normal diet, oral liquid diet for 24–48 h after surgery.
Postoperative PONV treatment	Patients regardless of their PONV risk should receive two or three antiemetic drugs for prophylaxis, such as dexamethasone, tropisetron, droperidol, and promethazine.
Psychological evaluation	Assess the patient's psychological condition; holistic care combined with psychological support care to improve the patient's mood.
Discharge	
Pain management	Oral non‐opioid medications only.
Diet	At least resume a semi‐liquid diet or oral nutritional supplements.
Ambulation	Ambulation with minimal assistance or independent ambulation.
Systemic audit	Routinely auditing and feedback are necessary for the implementation of ERAS protocols, maintaining high compliance to ERAS protocols, and realizing quality improvement.

### 3.4 Delphi Survey Process of SSC

**FIGURE 1:**
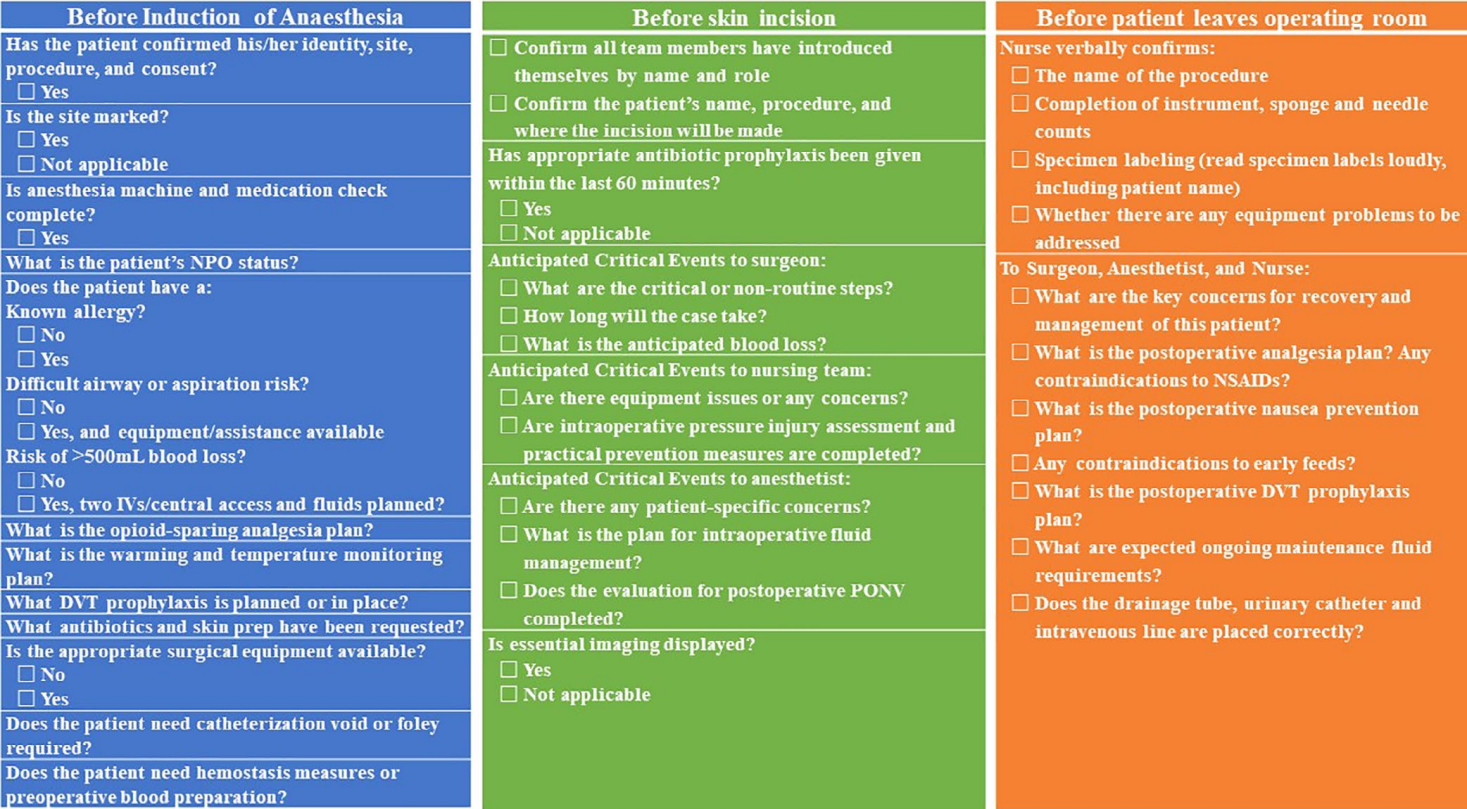
Detailed definitions and scheme of SSC.

The first round of the online modified Delphi survey was conducted from April 1 to April 15, 2024. The second round of the Delphi survey was conducted from May 1 to May 15, 2024. All participants (*n* = 75, 100%) completed the questionnaires in the two rounds. At the end of the process, a consensus was arrived at for 37 consolidated items in the following order of the domains: before induction of anesthesia, before skin incision and before the patient leaves the operating room. The complete SSC assessment process is shown in Table 3. The final SSC is shown in Figure 1.

**TABLE 3:** Summary of the Delphi surveys process of SSC.

SSC items	Delphi round 1	Delphi round 2	Consensus meeting
Before induction of anesthesia			
Has the patient confirmed his/her identity, site, procedure, and consent?	Include	Include	Include
Is the site marked?	Include	Include	Include
Is anesthesia machine and medication check complete?	Include	Include	Include
What is the patient's NPO status?	Include	Include	Include
Known allergy?	Include	Include	Include
Difficult airway or aspiration risk?	Include	Include	Include
Risk of > 500 mL blood loss?	Include	Include	Include
What is the opioid‐sparing analgesia plan?	Include	Include	Include
What is the warming and temperature monitoring plan?	Include	Include	Include
What DVT prophylaxis is planned or in place?	Include	Include	Include
What antibiotics and skin prep have been requested?	Include	Include	Include
Is the appropriate surgical equipment available?	Include	Include	Include
Does the patient need catheterization void or Foley required?	Include	Include	Include
Does the patient need hemostasis measures or preoperative blood preparation?	Include	Include	Include
Before skin incision			
Confirm all team members have introduced themselves by name and role.	Include	Include	Include
Confirm the patient's name, procedure, and where the incision will be made.	Include	Include	Include
Has appropriate antibiotic prophylaxis been given within the last 60 min?	Include	Include	Include
What are the critical or non‐routine steps?	Include	Include	Include
How long will the case take?	Include	Include	Include
What is the anticipated blood loss?	Include	Include	Include
Are there equipment issues or any concerns?	Include	Include	Include
Are intraoperative pressure injury assessment and practical prevention measures are completed?	Include	Include	Include
Are there any patient‐specific concerns?	Include	Include	Include
What is the plan for intraoperative fluid management?	Include	Include	Include
Does the evaluation for postoperative PONV completed?	Include	Include	Include
Is essential imaging displayed?	Include	Include	Include
Before the patient leaves the operating room			
The name of the procedure	Include	Include	Include
Completion of instrument, sponge, and needle counts	Include	Include	Include
Specimen labeling (read specimen labels loudly, including patient name)	Include	Include	Include
Whether there are any equipment problems to be addressed	Include	Include	Include
What are the key concerns for the recovery and management of this patient?	Include	Include	Include
What is the postoperative analgesia plan? Any contraindications to NSAIDs?	Include	Include	Include
What is the postoperative nausea prevention plan?	Include	Include	Include
Any contraindications to early feeds?	Include	Include	Include
What is the postoperative DVT prophylaxis plan?	Include	Include	Include
What are expected ongoing maintenance fluid requirements?	Include	Include	Include
Does the drainage tube, urinary catheter, and intravenous line are placed correctly?	Include	Include	Include

## 4 Discussion

In this study, we used a modified Delphi method to discuss the ERAS protocol for SM surgery, synthesizing findings from previous research. We obtained 37 consensus recommendations related to ERAS. Additionally, we developed an SSC specifically for SM surgery within the ERAS framework, which we also validated using the Delphi method. Given the lack of established ERAS protocols for SM surgery, our consensus statements and SSC provide a valuable framework for standardizing the ERAS protocols. We hope our findings will lay the foundation for the further refinement of ERAS protocols in the context of SM surgery.

### 4.1 Consensus Statements Regarding Preoperative Counseling and Education

To date, no study has directly indicated the effect of preoperative education on the surgical outcomes of SM. Nevertheless, our study respondents also reached a consensus that preoperative counseling and education should be recommended in the ERAS protocols. Research has shown that educational interventions significantly reduce the LOS in ERAS programs for colorectal surgery [29, 30]. Comprehensive preoperative education and counseling by clinical physicians, anesthesiologists, and nursing staff were considered valuable inputs. Patients who receive detailed education and counseling regarding anesthetization methods, extubation processes, and expected responses during extubation exhibit improved readiness for extubation, enhanced extubation quality, and better recovery following general anesthetization [31]. Moreover, thorough preoperative education was shown to reduce patient anxiety and improve adherence to postoperative treatment plans, thereby minimizing perioperative complications in patients [32, 33]. Similar benefits might be observed in the context of SM surgery. However, further extensive randomized controlled trials are required to confirm the effects of preoperative counseling on patient expectations and surgical outcomes in patients undergoing SM procedures. Following a Delphi discussion, preoperative postural training was excluded from the study protocol. Nevertheless, some experts involved in the discussion suggest its potential integration into preoperative patient education. The current study does not provide definitive evidence regarding the impact of preoperative postural training on the outcomes of SM surgery. However, some research indicates that preoperative adaptive training, such as tracheal lateral push and prone position exercises, may facilitate anesthesia extubation and enhance intraoperative hemodynamic stability. The lack of robust evidence precludes its adoption as a standard element in ERAS protocols. This training regimen also encompasses respiratory function exercises, including techniques for backslapping, coughing, and expectoration.

### 4.2 Consensus Statements Regarding Nutritional Status

Malnutrition is prevalent among patients with cancer and is often overlooked by clinicians. It is associated with increased LOS and morbidity following spinal surgery. Preoperative nutritional counseling, along with the monitoring of body mass index and serum albumin levels, constitutes basic assessment methods. The evaluation of preoperative malnutrition can be further enhanced using laboratory tests, physical measurements, and nutritional scoring systems, such as the Prognostic Nutritional Index (PNI). The interactions and prognostic significance of various nutritional and frailty indicators in patients with SM were examined in a study. Compared with the preoperative BMI, albumin levels, albumin‐to‐globulin ratio, and platelet‐to‐lymphocyte ratio of the patients, the PNI was identified as the most effective predictor of complications and a potential critical biomarker for risk stratification when assessed within 90 days postoperatively [34]. This study reached a consensus recommending a comprehensive nutritional assessment before surgery, in collaboration with specialized nutrition departments, to reduce postoperative complications.

### 4.3 Consensus Statements Regarding Patient Evaluation

Perioperative assessment is crucial for patients with SM. Given the variability in the preoperative physical status of individuals, rigorous evaluation using standardized scales is essential for adjusting the conditions of patients to an appropriate surgical baseline. In the ERAS protocol, the timing of application aligns with conventional surgical practices. The consensus did not endorse any specific scale or scoring system. Instead, it suggested that users of the ERAS protocol should select an appropriate scheme in need. Implementing patient assessments at key time points throughout the ERAS process was also recommended, along with continuous follow‐up throughout the treatment cycle. This approach helps enhance the understanding of the long‐term effects of ERAS on patient survival.

Moreover, the respondents unanimously reached a consensus that all hospitalized patients should undergo VTE risk assessment. Experimental data demonstrated a 6.3% incidence of deep vein thrombosis in patients undergoing decompression and internal fixation for SM, with elevated postoperative D‐dimer levels on the first day post‐surgery correlating with a greater risk of deep vein thrombosis. Patients requiring surgical intervention for SM represent a unique population that is at risk for venous thromboembolism. Understanding thrombotic conditions at multiple preoperative and postoperative stages may provide opportunities for early intervention and risk stratification in critically ill patient populations.

The decision‐making process in SM treatment is complex owing to various pathogenic factors, unpredictable expected survival, difficulty in quantifying preoperative factors, and rapid alterations in the clinical status of patients. The trend in SM management tended toward the integrated application of multiple therapeutic modalities, with some scholars advocating for a multidisciplinary, collaborative, multimodal comprehensive treatment system. In this study, the respondents agreed that selecting an appropriate prognostic scoring system during the diagnostic and therapeutic processes was important.

### 4.4 Consensus Statements Regarding Psychological Evaluation

The consensus recommended the inclusion of scales that assess anxiety and depression; 24.0% of respondents suggested that “psychological evaluation” should be added to the ERAS program. Findings from numerous studies have indicated that anxiety and depression are associated with cancer recurrence, and the cancer status could exacerbate symptoms of anxiety and depression in patients. Preoperative anxiety and depression are correlated with increased pain, impaired physical function, and diminished health‐related quality of life in patients undergoing spinal surgery. Therefore, we hypothesized that assessing and adjusting the psychological states of patients may improve surgical outcomes for patients with SM. Commonly used psychological assessment tools for patients with cancer include the State–Trait Anxiety Inventory, the Hospital Anxiety and Depression Scale (HADS), the Hamilton Anxiety Scale, and various self‐developed scales. A study on 2121 consecutive adults inpatients with cancer used the HADS, the state anxiety subscale of the State–Trait Anxiety Inventory, and the Center for Epidemiologic Studies Depression Scale. The findings indicated that high‐risk scores for anxiety and depression, which exceed specific HADS threshold values, could help identify anxiety and depression in patients with cancer. Evidence from systematic reviews demonstrates that interventions such as relaxation training, cognitive behavioral therapy, hypnosis, emotional counseling, and integrative psychotherapy positively influence anxiety experienced by patients during the acute postoperative period and enhance the psychological components of quality of life in long‐term follow‐ups following orthopedic surgery. Additionally, in a systematic review, the effectiveness of psychosocial approaches in adult orthopedic surgery was assessed. Notably, psychosocial interventions, particularly patient education and relaxation techniques, exhibited significant efficacy in alleviating postoperative pain in patients.

### 4.5 Consensus Statements Regarding Antithrombotic Prophylaxis

Patients undergoing orthopedic surgery are at a higher risk of thromboembolic events. Preventive measures for thrombosis include early mobilization, mechanical prophylaxis (such as the use of graduated compression stockings, intermittent pneumatic compression of the lower limbs, foot compression devices, and intermittent pneumatic compression of the upper limbs), and pharmacological prophylaxis with low‐molecular‐weight heparin. Although there is no evidence indicating a higher risk of thromboembolism in patients undergoing surgery for SM at present, the study findings suggested that low‐molecular‐weight heparin was safe for these patients. Additionally, prolonged postoperative bed rest was a significant independent risk factor for venous thromboembolism. Facilitating early mobilization for patients aligns with the recommendation of the ERAS protocol developed in our study, which could help potentially reduce the incidence of venous thromboembolism.

### 4.6 Consensus Statements Regarding Temperature Management

In this study, 29.3% of the respondents suggested that “temperature management” should be added to the ERAS program. Perioperative accidental hypothermia can occur owing to the suppression of thermoregulatory central mechanisms under anesthesia and the prolonged exposure of large areas of the skin to the low temperatures in the operating room. Such cases of unintended hypothermia during the perioperative period are associated with clinical complications, including surgical site infections, delayed wound healing, increased bleeding, and cardiovascular events. Patients undergoing general anesthesia for more than 30 min, especially patients undergoing major surgeries, should have their body temperature monitored and maintained. We recommend continuous temperature monitoring throughout the surgical procedure and the proactive application of various warming or heating methods to maintain the body temperature of patients above 36°C. Research indicated that active preoperative or intraoperative surface warming reduces the incidence of surgical site infections and decreases intraoperative blood loss. Another study demonstrated that active warming using forced air heaters, intravenous fluid warmers, and heated beds in patients undergoing elective surgery for scoliosis correction significantly reduced intraoperative blood loss, reduced the duration of surgery, and decreased hospital stay in patients. To date, no study has reported the negative impacts of intraoperative active warming or thermal maintenance systems on surgeries for SM.

### 4.7 Consensus Statements Regarding Anesthesia Management

The respondents reached a consensus on the choice of general anesthesia as the preferred method of anesthetization for SM resection surgeries. With respect to anesthetic drug selection, ERAS protocols aim to minimize or eliminate the use of intraoperative opioids. A study in which lidocaine and dexmedetomidine were used as substitutes for opioid infusion reported a significant reduction in postoperative opioid consumption [35]. Another study in which an ERAS protocol with Opioid‐Free Anesthesia (OFA) in lumbar decompression surgery was reported indicated that total perioperative opioid consumption was significantly decreased when the OFA approach was followed [36].

Additionally, research suggested the use of spinal anesthesia in elderly patients with American Society of Anesthesiologists scores of 3 or 4, who face greater risks associated with general anesthesia. However, the applicability of this approach was limited, and its widespread safety was not supported by sufficient robust evidence. Therefore, it was recommended primarily as an alternative when general anesthesia was not feasible.

Furthermore, the consensus suggested that ERAS protocols should incorporate the local administration of anesthetics to enhance pain management. Reports recommended the optimal combination of levobupivacaine (200 mg/100 mL, 0.9% saline), ketorolac (30 mg), and epinephrine (0.5 mg) as a wound infiltration agent. Effective local infiltration could reduce the postoperative analgesic requirements and expedite discharge. The addition of local anesthetics, along with various adjunctive medications, such as dexamethasone and dexmedetomidine, reportedly improved and prolonged the duration of analgesia.

### 4.8 Consensus Statements Regarding TCM and TCM Therapy

In the surgical treatment of SM, TCM plays a role in several key areas. TCM can enhance the quality of life of patients with cancer. Herbal medicine therapies are highly valuable for enhancing impaired immune function, particularly in cases of malnutrition and chronic diseases [37]. These herbs have demonstrated a significant ability to inhibit cell viability and proliferation, promote apoptosis, and reduce matrix metalloproteinases (MMPs), thereby inducing apoptosis in cancer cells [38]. Currently, TCM plays a role in modulating both innate and adaptive immunity in the context of cancer. Integrating immune‐enhancing herbal medicines with conventional therapies presents a promising clinical strategy. For instance, polysaccharides derived from TCM, as naturally occurring bioactive macromolecules, exhibit antitumor effects by enhancing immune regulation [39]. Compared with traditional antitumor treatments, TCM polysaccharides offer the advantage of acting through multiple pathways and targeting various molecular sites [40].

Herbal medicine could improve the appetite of patients, thereby enhancing their nutritional status, and it has applications in post‐surgery, post‐chemotherapy, and/or post‐radiotherapy recovery. TCM offers distinct advantages in mitigating the side effects of radiotherapy, enhancing the sensitivity of tumor tissues to radiation, and improving therapeutic outcomes. The combination of chemotherapy and TCM can effectively reduce toxicity, decrease the incidence of severe vomiting, and alleviate bone marrow suppression. Furthermore, the integration of TCM with targeted therapies can effectively delay the development of drug resistance, enhance patients' quality of life, and broaden the applicability of targeted treatments [41]. Cancer‐related fatigue was defined as a feeling of tiredness or exhaustion associated with cancer or its treatment. Certain herbal medicines can improve nighttime sleep and alleviate fatigue symptoms in patients.

TCM can also help reduce the incidence of postoperative complications, such as postoperative bowel obstruction. The therapeutic effects of TCM on common postoperative complications, such as bowel obstruction in orthopedic surgeries, were also observed, with various herbal medicines effectively alleviating pain, bloating, and fecal retention. TCM offers individualized treatment for infectious diseases, including bacterial, fungal, and viral infections, demonstrating clear efficacy and distinct advantages. The treatment modalities include the use of single herbs, herbal formulas, individual herbal compounds, or even combinations with antibiotics. This approach ensures anti‐inflammatory effects while reducing the occurrence of bacterial resistance [42]. A study on the use of TCM after colorectal cancer surgery revealed that the TCM treatment group had a significantly lower incidence of postoperative wound infections, reduced LOS, and a notable decrease in antibiotic use [43].

The dependence of comprehensive tumor treatment on multidisciplinary consultations is increasing. The involvement of TCM represents a significant advantage in cancer treatment in China. The consensus recommended the treatment of bloating and constipation and external use of Chinese medicine and fumigation therapy (42.7% of respondents suggested that “treatment of bloating and constipation and external use of Chinese medicine and fumigation therapy” should be added to the ERAS program).

### 4.9 Consensus Statements Regarding SSC

The SSC is a well‐established tool aimed at optimizing perioperative patient outcomes. It involves the participation of surgeons, anesthesiologists, nurses, technicians, and other personnel involved in surgical procedures. Clinical evidence demonstrates that each safety verification measure included in the SSC can significantly reduce the likelihood of severe, preventable surgical injuries. The principles and functions of the SSC are closely aligned with those of the ERAS protocols. The checklist should not merely be perceived as a list of items to be verified; rather, it should be used as a tool for enhancing communication in the operating room, fostering teamwork, and promoting a culture of safety, and should be implemented accordingly.

The integration of the SSC with ERAS protocols for colorectal and gynecological oncology surgeries has been demonstrated previously. In this study, we also explored the WHO SSC to develop an SSC tailored for SM surgery. This SSC can closely align with certain components of the ERAS protocol. It required surgeons and anesthesiologists to establish appropriate analgesic plans preoperatively while ensuring that all surgical team members are aware of the risks of perioperative deep vein thrombosis. Furthermore, it emphasized the importance of anticipating perioperative risks, including bleeding, infection, and pressure injuries, and the proper placement of various catheters, thereby maximizing the rigor and benefits of the ERAS protocol.

However, it is equally important to note that the effective application of the SSC is contingent upon compliance. Despite our efforts to optimize certain items of the WHO SSC through a consultative process, we cannot guarantee its applicability to all medical teams, and the complexity of the SSC process might increase the workload of surgical team members. Therefore, consensus recommendations regarding the SSC should be promoted only after its compliance is confirmed and appropriate adjustments are made.

## 5 Limitations and Strengths

The study presented an ERAS protocol and SSC plan for SM developed through a comprehensive Delphi process. This approach prioritized clinical efficacy over‐reliance on a single metric or score. Notably, this investigation was the first to integrate TCM and SSC into an ERAS protocol, thereby establishing a standardized framework for this approach and demonstrating the feasibility of applying the revised SSC protocol to ERAS in SM surgery. This study served as a valuable resource for evaluating the quality of subsequent related research and identifying potential gaps in complementary studies.

The Delphi study has inherent limitations. The consensus process, based on expert opinions, integrates existing evidence, with summaries of the current literature included as a part of the preliminary investigation. However, the outcome was primarily grounded in the cumulative experience of the group. The recommendations derived from the study lack empirical clinical support, necessitating further clinical research to assess their efficacy and adherence.

The heterogeneity of SM, both in terms of histology and location, poses a significant challenge in the standardization of ERAS protocols. For instance, lesions involving the spinal cord may require more intensive perioperative monitoring and management than those located extramedullary. Furthermore, the complexity of SM surgeries, which often require multidisciplinary teams and specialized equipment, may complicate the consistent application of ERAS principles across institutions. This is particularly true in resource‐limited settings, where access to specialized care may be constrained. The conclusions drawn from the study might only be applicable to specific regions. We recommend that different centers customize their ERAS protocols according to the unique circumstances.

## 6 Conclusions

This study, based on the Delphi method, yielded 37 consensus‐driven best practice recommendations, including suggestions related to perioperative anesthesia, surgery, and nursing for SM. We hope that this study, in which we integrated both TCM and Western medical treatment protocols, could form a basis for the establishment of a rapid rehabilitation program for surgical intervention in SM.

## Author Contributions

F.L., Y.J., and Y.W. initiated the collaborative project. H.Z., F.Z., and Z.Z. participated in the conceptualization. Q.G., P.C., and X.G. participated in manuscript preparation and review of the manuscript in several stages. M.Y. and P.C. approved the final version of the manuscript and the revisions as needed. The guarantor of the study is Q.H., X.Y., P.C., and M.Y.; accepts full responsibility for the finished work and/or the conduct of the study, had access to the data, and controlled the decision to publish.

## Consent

The authors have nothing to report.

## Conflicts of Interest

The authors declare no conflicts of interest.

## Supporting information


**Table S1.** The occupation, workplace, and city of the participants.

## Data Availability

All supporting data are available from the corresponding author upon reasonable request.
