# Supplementary material for: Construction and Development of an Enhanced Recovery After Surgery Program for the Surgical Management of Patients With Spinal Metastasis: A Modified Delphi Study
Source: Orthop Surg. 2025 Jan 23;17(3):939–52. doi: 10.1111/os.14375 (PMC11872384; doi:10.1111/os.14375)
Supplement: Supplementary file 1 — Table S1. The occupation, workplace, and city of the participants. [file OS-17-939-s001.docx]

**Table 1s. The occupation, workplace and city of the participants.**

| **Multidisciplinary** | **No** | **Name** | **Institution** | **Province/City** | **Degree/Post** |
| --- | --- | --- | --- | --- | --- |
| **Clinical Workers** | 1 | Mengchen Yin | Longhua Hospital | Shanghai | Senior |
|  | 2 | Junming Ma | Longhua Hospital | Shanghai | Senior |
|  | 3 | Wenlong Yu | Longhua Hospital | Shanghai | Senior |
|  | 4 | Xinghai Yang | Changzheng Hospital | Shanghai | Senior |
|  | 5 | Quan Huang | Changzheng Hospital | Shanghai | Senior |
|  | 6 | Minglei Yang | Changzheng Hospital | Shanghai | Senior |
|  | 7 | Luosheng Zhang | Changzheng Hospital | Shanghai | Intermediate |
|  | 8 | Dingbang Chen | Changzheng Hospital | Shanghai | Intermediate |
|  | 9 | Tao Wang | The Second Hospital of Anhui Medical University | Anhui | Intermediate |
|  | 10 | Shibing Zhao | The Second Hospital of Anhui Medical University | Anhui | Intermediate |
|  | 11 | Yanxin Liu | The Second Hospital of Anhui Medical University | Anhui | Intermediate |
|  | 12 | Zhengwang Sun | Shanghai Cancer Center | Shanghai | Senior |
|  | 13 | Wangjun Yan | Shanghai Cancer Center | Shanghai | Senior |
|  | 14 | Mo Cheng | Shanghai Cancer Center | Shanghai | Senior |
|  | 15 | Dong Wang | Hangzhou Hospital of Traditional Chinese Medicine | Zhejiang | Senior |
|  | 16 | Hongshen Wang | No.2 Hospital of Guangzhou Traditional Chinese Medicine | Guangzhou | Senior |
|  | 17 | Yongping Lin | No.2 Hospital of Guangzhou Traditional Chinese Medicine | Guangzhou | Senior |
|  | 18 | Xudong Wang | 905 Hospital | Shanghai | Senior |
|  | 19 | Liangliang Gao | 905 Hospital | Shanghai | Senior |
|  | 20 | Jie Zhang | 905 Hospital | Shanghai | Intermediate |
| **Researchers** | 1 | Xing Ding | Shanghai University of Traditional Chinese Medicine | Shanghai | Intermediate |
|  | 2 | Chongqing Xu | Shanghai University of Traditional Chinese Medicine | Shanghai | Intermediate |
|  | 3 | Yongjia Yi | Shanghai University of Traditional Chinese Medicine | Shanghai | Intermediate |
|  | 4 | Wenhao Zhu | Shanghai University of Traditional Chinese Medicine | Shanghai | Intermediate |
|  | 5 | Yu Xiao | Shanghai University of Traditional Chinese Medicine | Shanghai | Intermediate |
|  | 6 | Yinjie Yan | Longhua Hospital | Shanghai | Senior |
|  | 7 | Tao Wang | Longhua Hospital | Shanghai | Senior |
|  | 8 | Zhaoxiang Fan | Longhua Hospital | Shanghai | Senior |
|  | 9 | Fanjie Meng | Longhua Hospital | Shanghai | Intermediate |
|  | 10 | Xuanyu Wang | Longhua Hospital | Shanghai | Intermediate |
|  | 11 | Wei Chen | Shanghai Tonghe Orthopedic Hospital | Shanghai | Intermediate |
|  | 12 | Yanqiang Wei | Shanghai Tonghe Orthopedic Hospital | Shanghai | Intermediate |
|  | 13 | Xiaotao Xie | Shanghai Tonghe Orthopedic Hospital | Shanghai | Intermediate |
|  | 14 | Baohang Reng | Shanghai Tonghe Orthopedic Hospital | Shanghai | Intermediate |
|  | 15 | Shan He | Shanghai Tonghe Orthopedic Hospital | Shanghai | Intermediate |
| **Anesthesiologists** | 1 | Xin Jiang | Changzheng Hospital | Shanghai | Senior |
|  | 2 | Shan Xu | Changzheng Hospital | Shanghai | Senior |
|  | 3 | Shuhan Zhang | Changzheng Hospital | Shanghai | Senior |
|  | 4 | Dongyu Zheng | Changzheng Hospital | Shanghai | Senior |
|  | 5 | Xin Gao | Changzheng Hospital | Shanghai | Senior |
|  | 6 | Zihuan Zhou | Changzheng Hospital | Shanghai | Senior |
|  | 7 | Haohan Zhou | Changzheng Hospital | Shanghai | Senior |
|  | 8 | Tao Li | Maanshan General Hospital of Ranger-Duree Healthcare | Anhui | Senior |
|  | 9 | Chengchun Jin | Maanshan General Hospital of Ranger-Duree Healthcare | Anhui | Senior |
|  | 10 | Peilin Chu | Maanshan General Hospital of Ranger-Duree Healthcare | Anhui | Senior |
|  | 11 | Qiang Gao | Maanshan General Hospital of Ranger-Duree Healthcare | Anhui | Senior |
|  | 12 | Fanjie Li | Maanshan General Hospital of Ranger-Duree Healthcare | Anhui | Intermediate |
|  | 13 | Qinghua Zhang | Longhua Hospital | Shanghai | Senior |
|  | 14 | Zhen Ma | Longhua Hospital | Shanghai | Senior |
|  | 15 | Qing Luo | Longhua Hospital | Shanghai | Senior |
| **Nursing Specialist** | 1 | Changli Wan | Changzheng Hospital | Shanghai | Senior |
|  | 2 | Xiaolin Li | Changzheng Hospital | Shanghai | Senior |
|  | 3 | Xuemei Huang | Changzheng Hospital | Shanghai | Senior |
|  | 4 | Minzhen Liao | Changzheng Hospital | Shanghai | Senior |
|  | 5 | Tingtin Bai | Changzheng Hospital | Shanghai | Senior |
|  | 6 | Xueting Wang | Changzheng Hospital | Shanghai | Senior |
|  | 7 | Jin Lv | Changzheng Hospital | Shanghai | Intermediate |
|  | 8 | Zhihao Liang | Longhua Hospital | Shanghai | Intermediate |
|  | 9 | Qun Li | Longhua Hospital | Shanghai | Intermediate |
|  | 10 | Junning Zhou | Longhua Hospital | Shanghai | Intermediate |
|  | 11 | Yiwen Xu | Longhua Hospital | Shanghai | Intermediate |
|  | 12 | Liwen Wang | Longhua Hospital | Shanghai | Intermediate |
|  | 13 | Ping Li | Longhua Hospital | Shanghai | Intermediate |
|  | 14 | Nannan Dai | Longhua Hospital | Shanghai | Intermediate |
|  | 15 | Jie Xu | Longhua Hospital | Shanghai | Intermediate |
| **Psychologists** | 1 | Mingwei Song | Yongci Rehabilitation Hospital | Shanghai | Senior |
|  | 2 | Hailiang Li | Yongci Rehabilitation Hospital | Shanghai | Senior |
|  | 3 | Pengbo Reng | Yongci Rehabilitation Hospital | Shanghai | Senior |
|  | 4 | Panpan Wang | Yongci Rehabilitation Hospital | Shanghai | Intermediate |
|  | 5 | Shu Zhao | Yongci Rehabilitation Hospital | Shanghai | Intermediate |
| **caregivers** | 1 | Yang | Changzheng Hospital | Shanghai | N/A |
|  | 2 | Shi | Changzheng Hospital | Shanghai | N/A |
|  | 3 | Zhang | Changzheng Hospital | Shanghai | N/A |
|  | 4 | She | Changzheng Hospital | Shanghai | N/A |
|  | 5 | Guo | Changzheng Hospital | Shanghai | N/A |
